# Cas9 Functionally Opens Chromatin

**DOI:** 10.1371/journal.pone.0152683

**Published:** 2016-03-31

**Authors:** Amira A. Barkal, Sharanya Srinivasan, Tatsunori Hashimoto, David K. Gifford, Richard I. Sherwood

**Affiliations:** 1 Division of Genetics, Department of Medicine, Brigham and Women’s Hospital and Harvard Medical School, Boston, Massachusetts, United States of America; 2 Computer Science and Artificial Intelligence Laboratory, Massachusetts Institute of Technology, Cambridge, Massachusetts, United States of America; 3 Department of Stem Cell and Regenerative Biology, Harvard University and Harvard Medical School, 7 Divinity Avenue, Cambridge, Massachusetts, United States of America; Osaka University, JAPAN

## Abstract

Using a nuclease-dead Cas9 mutant, we show that Cas9 reproducibly induces chromatin accessibility at previously inaccessible genomic loci. Cas9 chromatin opening is sufficient to enable adjacent binding and transcriptional activation by the settler transcription factor retinoic acid receptor at previously unbound motifs. Thus, we demonstrate a new use for Cas9 in increasing surrounding chromatin accessibility to alter local transcription factor binding.

## Introduction

Active gene regulatory elements are invariably found in regions of heightened chromatin accessibility, which are characterized by loosened contact between DNA and nucleosomes [1]. Regulation of chromatin accessibility plays a key role in determining where transcription factors (TFs) bind and which genes are active [2–5].

Chromatin accessibility is regulated in part by pioneer factors, a class of TFs which are capable of binding to inaccessible, nucleosome-bound DNA and inducing accessibility [4, 6, 7]. We recently characterized a chromatin-based transcription factor binding hierarchy in which pioneer factors open chromatin, enabling the adjacent binding of a distinct class of TFs, settler factors [5]. Settler factors appear to obey a simple rule in their binding: they bind when chromatin at their motif is accessible and do not bind when chromatin is inaccessible.

While previous work has uncovered the importance of chromatin accessibility in governing TF binding, it is unknown if altering chromatin accessibility is sufficient to alter settler factor binding. An alternative possibility is that changes in chromatin accessibility imply changes in other aspects of chromatin state such as histone modification [8] and DNA methylation [9] that may influence TF binding. Currently, there are no tools available to manipulate chromatin accessibility at native genomic loci, impeding our ability to ascertain causal relationships between chromatin accessibility and TF binding.

In this work, we assess the ability of the CRISPR/Cas (CRISPR) genome editing system to alter chromatin accessibility. In CRISPR, a short guide RNA (sgRNA) with sequence complementarity to a 17–20 base-pair (bp) genomic sequence complexes with the Cas9 protein to cleave DNA at that complementary genomic site, inducing local sequence mutants or enabling site-specific homologous recombination [10, 11]. Cas9 has been mutated to abolish its nuclease activity, creating a nuclease-dead Cas9 (dCas9), which binds site-specifically in the genome but does not cleave DNA [12]. dCas9 and fusion protein derivatives of it have been used in gene repression [12], gene activation (fused to a transcriptional activator domain) [12], locus-specific live imaging (fused to GFP) [13], and mass spectrometry of native gene regulatory elements [14].

In spite of the utility of dCas9 fusion proteins in a panoply of applications, it is unknown whether dCas9 by itself is able to induce chromatin accessibility after binding to previously inaccessible chromatin. If dCas9 is able to induce chromatin accessibility, it could be used to artificially open chromatin, permitting inquiry into the causal implications of chromatin accessibility and its role in affecting gene expression. The ability to focally open chromatin would be a step toward altering gene expression and cell fate predictably, allowing endogenous transcription factors and signaling pathways to populate the newly accessible chromatin. Additionally, if dCas9 opens chromatin, it may disrupt the native gene regulatory architecture at targeted loci, which may lead to erroneous results in CRISPR-based live imaging and mass spectrometry approaches. Lastly, determining whether dCas9 is able to open previously inaccessible chromatin is important in determining how likely it is to function when targeted to these inaccessible regions in the genome.

## Materials and Methods

### Cell culture

Mouse embryonic stem cell (mESC) culture was performed according to previously published protocols [5]. Undifferentiated 129P2/OlaHsd mESC were maintained on gelatin-coated plates feeder-free in mES media composed of Knockout DMEM (Life Technologies) supplemented with 15% defined fetal bovine serum (FBS) (HyClone), 0.1mM nonessential amino acids (Life Technologies), Glutamax (Life Technologies), 0.55mM 2-mercaptoethanol (Sigma), 1X ESGRO LIF (Millipore), 5 nM GSK-3 inhibitor XV and 500 nM UO126. Cells were regularly tested for mycoplasma. Genetic manipulations to stem cell lines are described below.

### Cell line generation

The Eco0109I-XbaI fragment from pX330 [10] was cloned into p2TAL200R175 [15, 16] and modified to introduce the FE mutations shown to improve guide RNA functionality [13]. A Hygromycin resistance cassette was added between the Tol2 sites to allow selection for genomically integrated lines. Specific guide RNAs were cloned into this vector using BbsI, and sgRNA sequences are listed in S1 Table. dCas9 was cloned from pAC91-pmax-dCas9VP64 [12] into p2TAL200R175 [15, 16], and a Blasticidin resistance cassette was added between the Tol2 sites to allow selection for genomically integrated lines. Tol2-containing reporter plasmids and transposase-containing pCAGGS-mT2TP [15, 16] were transfected into the mESC using Lipofectamine 3000 (Life Technologies). Hygromycin and/or Blasticidin selection were performed for >7 days in mES media.

### DNase hypersensitivity analysis

DNase hypersensitivity analysis was performed as described previously [5]. 10–100 million cells were digested with 60–100 units of DNase I (Promega) per 10^7^ nuclei. 50–125 bp hypersensitive DNA was collected using E-Gel SizeSelect Agarose 2% gels (Life Technologies). qPCR was performed with Dynamo Flash SYBR Green qPCR kit (Thermo Scientific) using positive and negative control primers to normalize for variability in signal-to-noise of the DNase hypersensitivity assay. All experiments used in this paper had >20-fold enrichment of positive over negative control primers. Primer sequences used in this work are listed in S1 Table.

### Chromatin immunoprecipitation

Chromatin immunoprecipitation was performed according to the “Mammalian ChIP-on-chip” protocol (Agilent) using a polyclonal antibody against retinoic acid receptor that recognizes all protein isoforms (M-454, Santa Cruz Biotechnology) and Protein G Dynabeads (Life Technologies). 10–100 million cells were used for each experiment. qPCR using positive and negative control primers was performed to ensure ChIP enrichment, and all experiments used in this paper had >10-fold enrichment. Primer sequences used in this work are listed in S1 Table.

### Tol2 RAR-GFP reporter

mESCs with a Tol2 transposon-integrated reporter construct containing 2x RAR binding site, minimal Hsp68 promoter, and GFP were generated previously [5]. CAGGS-dCas9 was added to these cells using the Tol2 transposon system as described above. sgRNAs were added to the cells when noted using the Tol2 transposon system. Primer sequences used to clone these sgRNAs are listed in S1 Table. Flow cytometry was performed using BD Accuri and data was analyzed on BD Accuri software.

## Results

**Fig 1:**
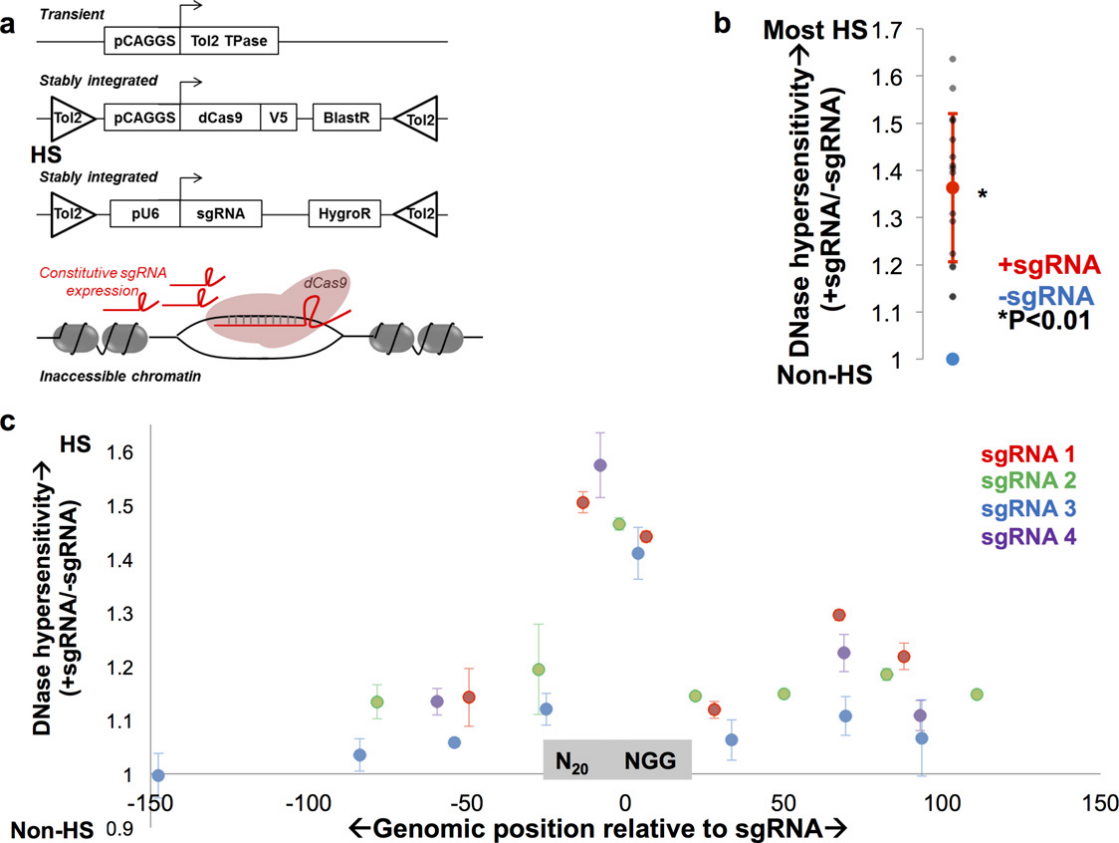
dCas9 induces chromatin accessibility at previously inaccessible genomic loci. **a**. mESCs were co-transfected with a Tol2 transposase (TPase), a Tol2 transposon-flanked dCas9 expression cassette, and a Tol2 transposon-flanked sgRNA cassette to yield stable expression of dCas9 and a sgRNA targeted to a region with inaccessible chromatin. **b**. 16/16 loci in previously inaccessible chromatin had statistically significant increases in DNase hypersensitivity (y-axis) upon sgRNA targeting as measured by DNase-qPCR (gray dots). DNase hypersensitivity at each locus is normalized to its level in the absence of sgRNA (blue dot), and the average normalized DNase hypersensitivity in the presence of gRNA for all loci is shown (red dot), which is statistically significantly increased over–sgRNA control. At least two replicates were performed for all conditions, and a two-tailed Student’s t-test used to calculate significance. **c**. DNase-qPCR measurement of DNase hypersensitivity (y-axis) is shown +/-150 bp from the sgRNA site (x-axis) at four targeted loci. DNase-qPCR values at each datapoint are normalized to hypersensitivity in the absence of sgRNA, and all loci are oriented such that the 20 bp sgRNA sequence is immediately to the left of 0 and the NGG PAM sequence is immediately to the right of 0. Three replicates were performed for all experiments.

To address whether dCas9 is capable of opening previously closed chromatin, we built two Tol2 transposon-based vectors [15, 17] allowing stable expression of dCas9 and sgRNAs through transposon integration in mouse embryonic stem cells (mESC) (Fig 1A). dCas9 was fused with a V5 epitope tag, allowing us to confirm persistent, strong, nuclear expression (S1 Fig), and sgRNAs were modified to increase stability [13].

Using this system, we targeted dCas9 to 16 genomic locations with minimal prior chromatin accessibility in mESC as measured by DNase hypersensitivity analysis [2, 3, 17, 18] (S2 Fig). None of these sites is designated as a DNase hypersensitive site, and none have hypersensitivity above the levels of typical heterochromatin sites in the genome. dCas9 increased DNase hypersensitivity around the sgRNA site to a statistically significant degree at 16/16 locations (Fig 1B). The degree of chromatin opening varied substantially, with some sgRNAs inducing equivalent hypersensitivity to the most hypersensitive genomic regions and other sgRNAs inducing a more modest increase in hypersensitivity (Fig 1B). Thus, in 16/16 previously closed chromatin loci, dCas9 targeting opens chromatin significantly.

To determine the spatial pattern of dCas9-induced chromatin opening, we assessed chromatin accessibility +/-150 bp from the sgRNA site at four previously closed loci. We found that dCas9 opens chromatin most strongly within +/-20 bp of the sgRNA (Fig 1C), yet accessibility is significantly higher than control at least +/-100 bp from the sgRNA in all four loci (Fig 1C). Thus, dCas9 produces a spike of hypersensitivity at the sgRNA site and modestly increases hypersensitivity at least in the surrounding 100 bp.

**Fig 2:**
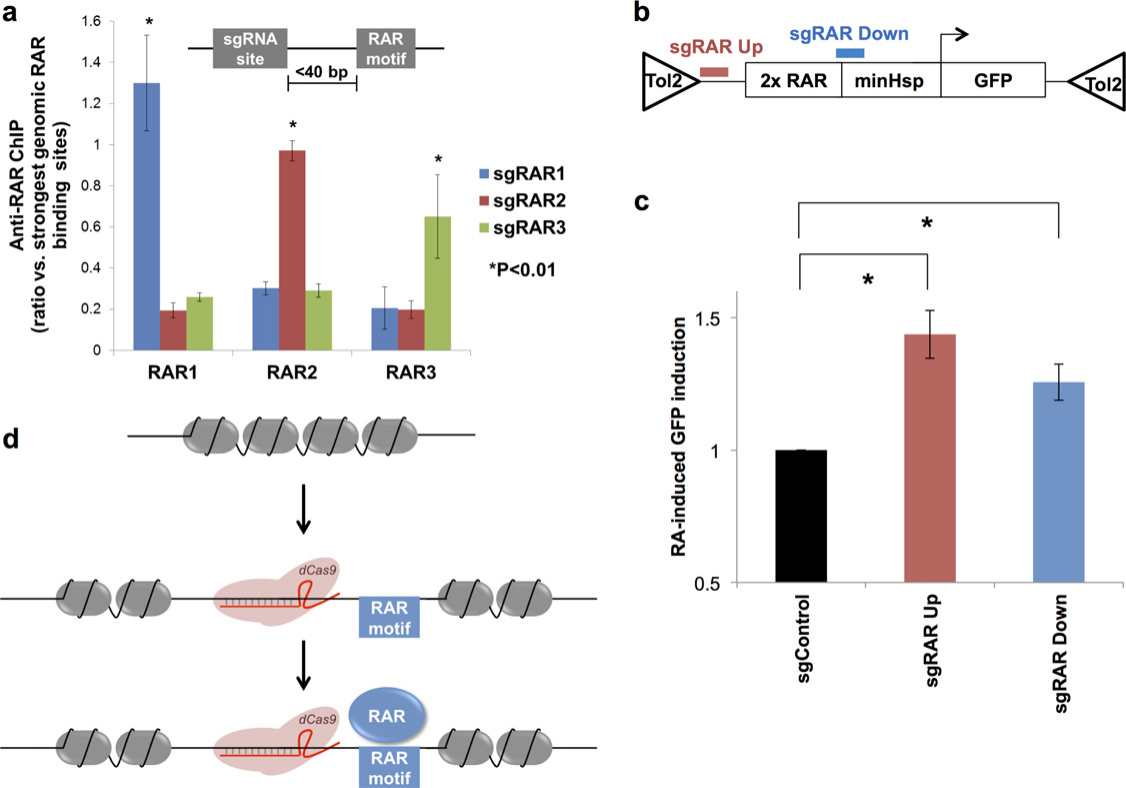
dCas9 chromatin opening enables adjacent RAR binding and RA-dependent gene activation. **a**. Anti-retinoic acid receptor (RAR) ChIP followed by qPCR at three loci (RAR1-3, x-axis) in the presence of sgRNAs targeting each locus (blue, red, and green). ChIP-qPCR values are normalized to the average of two of the strongest genomic RAR binding sites. Three replicates were performed for all experiments, and a two-tailed Student’s t-test was used to calculate significance, and values with P<0.01 are denoted with a *. **b.** The Tol2 transposon-based reporter system involves stable integration of a 2x RAR motif, a minimal promoter, and GFP. dCas9 was recruited through sgRNA sequences upstream (sgRAR Up, red) or downstream (sgRAR Down, blue) of the 2x RAR motif. **c**. Average flow cytometric GFP induction by RA in the presence of control sgRNA (black), sgRAR Up (red), or sgRAR Down (blue) sgRNAs. **d**. dCas9 is able to bind to sgRNA sequences in inaccessible chromatin and induce accessibility, which directly enables the settler factor RAR to bind to previously obscured motifs.

Is dCas9-induced chromatin accessibility functional? To assess whether dCas9-opened chromatin can facilitate the binding of a settler factor, we recruited dCas9 to three genomic loci within 40 bp of natively unbound motifs for the settler factor retinoic acid receptor (RAR) [5] (S3 Fig). We performed chromatin immunoprecipitation (ChIP) to compare RAR binding at each locus in the presence vs. absence of dCas9 recruitment. As expected, ChIP signal at all three loci was weak in the absence of dCas9 recruitment (Fig 2A, S4 Fig), and dCas9 targeting significantly increased ChIP signal at all three loci between 3 and 6-fold (Fig 2A, S4 Fig). In the presence of sgRNA, RAR binding was equivalent to binding at positive control sites representing some of the strongest genomic RAR binding sites (Fig 2A), indicating that dCas9-induced chromatin opening enables strong RAR binding at previously unbound sites.

We next asked whether the ability of dCas9 to alter local binding of RAR increases the ability of RAR to transcriptionally activate nearby genes. We used a GFP reporter system in which two strong RAR motifs are placed in front of a minimal promoter and GFP reporter gene and inserted into the genome using Tol2 transposon-based integration (Fig 2B). We have previously shown that addition of retinoic acid (RA) increases GFP expression poorly in this system by itself but increases GFP expression more robustly when local chromatin is made more accessible [5]. We recruited dCas9 either to a control location not present in the reporter DNA or to sites 10 bp upstream (SgRAR Up) or 20 bp downstream (SgRAR Down) of the RAR motifs in the reporter DNA and compared the ability of RA to induce GFP expression in these otherwise equivalent cell lines. We found that dCas9 recruitment either upstream or downstream of the RAR site significantly increases the ability of RA to induce GFP expression (Fig 2C). We note that dCas9 recruitment in the absence of RA does not influence GFP expression (S5 Fig). We thus conclude that dCas9 recruitment is sufficient to increase the transcriptional activation by RAR, presumably by increasing its local binding.

## Discussion

dCas9 consistently opens chromatin surrounding sgRNA sites. This opened chromatin enables binding and RA-dependent gene activation by the settler factor RAR at previously obscured motifs (Fig 2D). It is worth noting that chromatin is a complex, dynamic entity, and assays such as DNase hypersensitivity analysis collect average characteristics of thousands of cells. We do not know whether dCas9 displaces histones entirely as depicted in Fig 2D or merely decreases the strength or frequency of interactions between DNA and histones.

Since dCas9 is a bacterial protein, it is likely that its chromatin opening ability stems directly from its known ability to bind strongly to and mechanically unwind DNA [16,19,20]. It is unlikely that dCas9 interacts with eukaryotic chromatin or transcriptional machinery because it is foreign to mammalian cells, and existing evidence, although limited, does not show any effect of dCas9 alone on histone modifications [21, 22].

Our studies cannot distinguish whether it is the mechanical strand displacement and RNA-DNA heteroduplex formation [20] or the steric hindrance of nucleosomes by the bulky dCas9 that effect chromatin opening. dCas9-dependent chromatin opening consistently extends +/- 100 bp from its targeting site with a similar decay in magnitude over distance, suggesting that whatever the mechanism, it is consistent across target sites. Regardless, we show by the increase in RAR binding and gene activation nearby to dCas9-opened sites that dCas9’s effect is functionally opening chromatin and not merely increasing sensitivity to DNase I nuclease activity. An additional conclusion that follows from the use of a bacterial protein with no evolved chromatin modification activity to increase binding of the settler factor RAR is that such settler factor binding depends directly on the steric availability of the DNA at the motif site and not on protein-protein or protein-modified histone interactions. This conclusion provides direct evidence for the model that chromatin accessibility itself influences TF binding [5].

It is highly likely that dCas9 binding enables adjacent TF binding of many more TFs than the one we assayed, RAR. On the one hand, the fact that dCas9 disrupts the native transcription factor binding profile argues for caution in the interpretation of results from CRISPR-based approaches aimed at studying native genome function including those based on imaging and mass spectrometry, as dCas9 may disrupt the very events being measured. On the other hand, our demonstration that dCas9-induced chromatin opening can increase RA-induced gene expression introduces a paradigm of conditional gene activation dependent on the two independent parameters of dCas9 recruitment and RA administration, a form of transcriptional AND gating. Extended to other TFs, the ability of dCas9 to artificially induce chromatin accessibility will enable future studies of how TF binding and gene regulatory activities change at enhancers and promoters as chromatin becomes accessible and future work manipulating gene regulatory networks through rational alteration of chromatin accessibility.

## Supporting Information

S1 Fig Transposon-integrated mouse ESC express high levels of dCas9-V5. Immunofluorescence for V5 tag reveals strong, uniform, nuclear expression of dCas9-V5 after transposon integration (right panels) but not in wildtype mESC (left panels).
(PNG)

S2 Fig Guide RNA-targeted loci have minimal prior DNase hypersensitivity. DNase-seq signal in wildtype mESC (ENCODE ES-CJ7) in the 300 bp centered around the 16 guide RNA sites targeted in this work is uniformly weak, indicating that these regions are inaccessible in mESC. Reads per base are shown on the y-axis, and the 300 bp of genomic sequence surrounding the sgRNA sequences is shown on the x-axis. Rarb and Cyp26a1 loci, used as ChIP positive controls, are shown as comparisons that reside in accessible chromatin.
(PNG)

S3 Fig Guide RNA-targeted loci have minimal prior RAR ChIP signal. RAR ChIP signal in wildtype mESC (129P2/OlaHsd mESC used in this work) in the 300 bp regions surrounding the three RAR motif-adjacent sgRNAs targeted by dCas9. Reads per base are shown on the y-axis, and the 300 bp genomic regions centered around sgRNAs are shown on the x-axis. All three have minimal RAR ChIP prior to dCas9 recruitment. Rarb and Cyp26a1 are used in the DNase-qPCR analysis as positive controls, and ChIP signal at sgRar1-3 loci becomes equivalently strong to these loci after dCas9 recruitment.
(PNG)

S4 Fig dCas9 chromatin opening enables adjacent RAR binding. Anti-retinoic acid receptor (RAR) ChIP followed by qPCR at three loci (RAR1-3, x-axis) in the presence of sgRNAs targeting each locus (blue, red, and green). ChIP-qPCR values are normalized to control ChIP without sgRNA. Three replicates were performed for all experiments, and a two-tailed Student’s t-test was used to calculate significance, and values with P<0.01 are denoted with a *.
(PNG)

S5 Fig dCas9 alone does not affect reporter GFP expression. GFP expression in Tol2 RAR-GFP reporter cells in the absence of RA, normalized to cells without sgRNA. The presence of sgRNA recruiting dCas9 upstream (sgRAR up) or downstream (sgRAR down) of the RAR site has no measurable effect on GFP expression in the absence of RA.
(PNG)

S1 Table Oligonucleotides used in this work. (DOCX)
