# Supplementary figures and images for: Cas9 Functionally Opens Chromatin
Source: PLoS One. 2016 Mar 31;11(3):e0152683. doi: 10.1371/journal.pone.0152683 (PMC4816323; doi:10.1371/journal.pone.0152683)

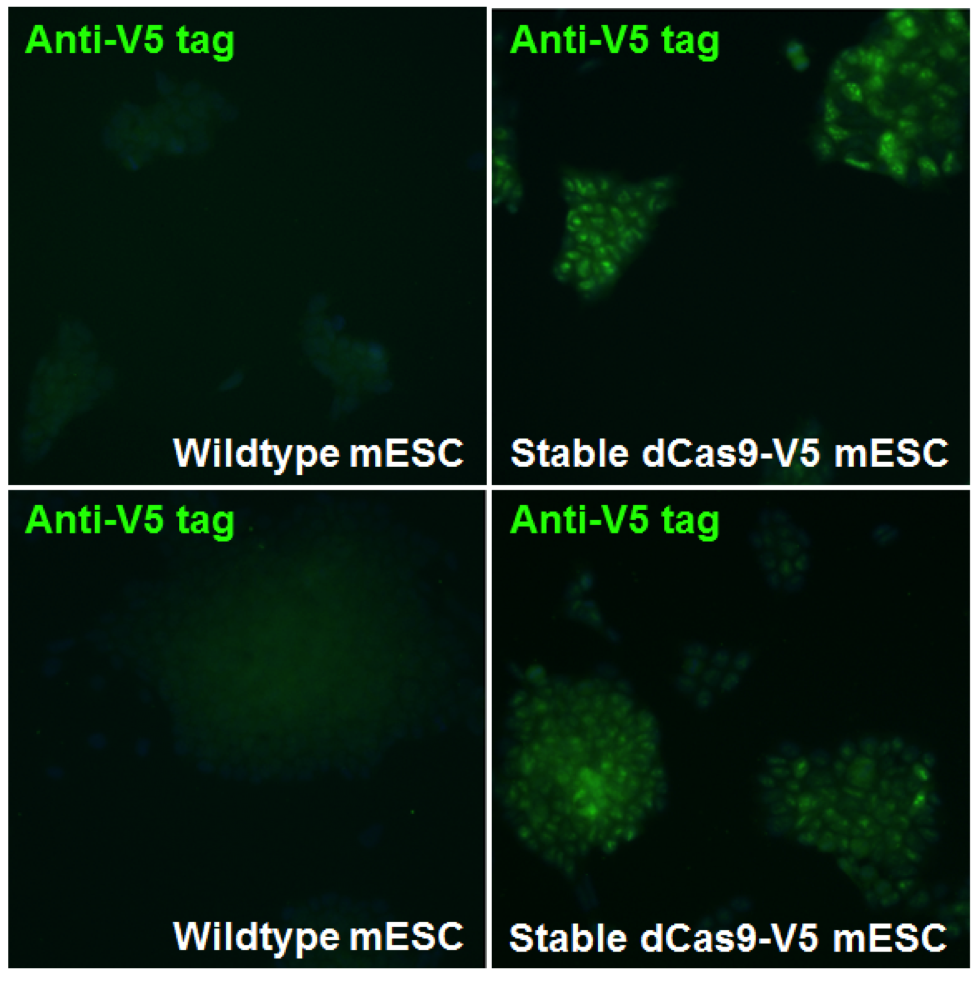

Supplement: S1 Fig — Immunofluorescence for V5 tag reveals strong, uniform, nuclear expression of dCas9-V5 after transposon integration (right panels) but not in wildtype mESC (left panels). (PNG) [file pone.0152683.s001.png]

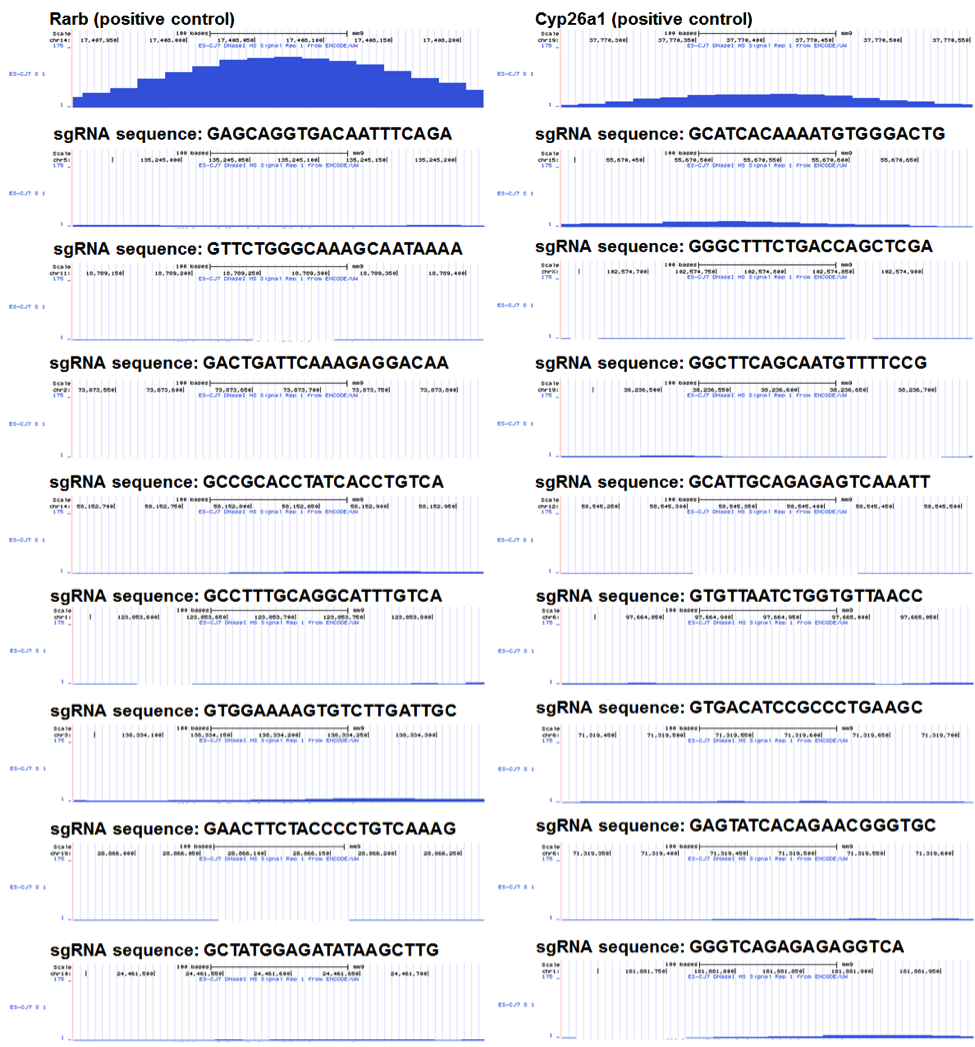

Supplement: S2 Fig — DNase-seq signal in wildtype mESC (ENCODE ES-CJ7) in the 300 bp centered around the 16 guide RNA sites targeted in this work is uniformly weak, indicating that these regions are inaccessible in mESC. Reads per base are shown on the y-axis, and the 300 bp of genomic sequence surrounding the sgRNA sequences is shown on the x-axis. Rarb and Cyp26a1 loci, used as ChIP positive controls, are shown as comparisons that reside in accessible chromatin. (PNG) [file pone.0152683.s002.png]

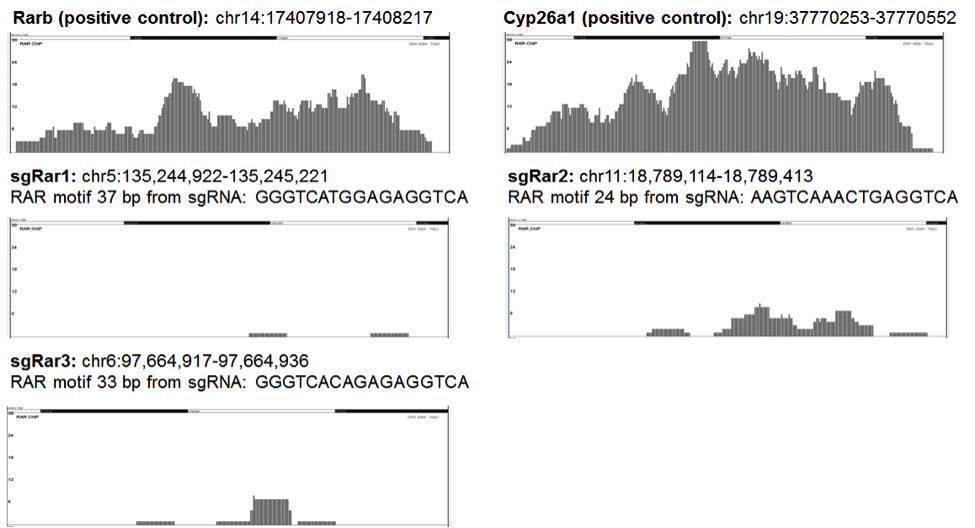

Supplement: S3 Fig — RAR ChIP signal in wildtype mESC (129P2/OlaHsd mESC used in this work) in the 300 bp regions surrounding the three RAR motif-adjacent sgRNAs targeted by dCas9. Reads per base are shown on the y-axis, and the 300 bp genomic regions centered around sgRNAs are shown on the x-axis. All three have minimal RAR ChIP prior to dCas9 recruitment. Rarb and Cyp26a1 are used in the DNase-qPCR analysis as positive controls, and ChIP signal at sgRar1-3 loci becomes equivalently strong to these loci after dCas9 recruitment. (PNG) [file pone.0152683.s003.png]

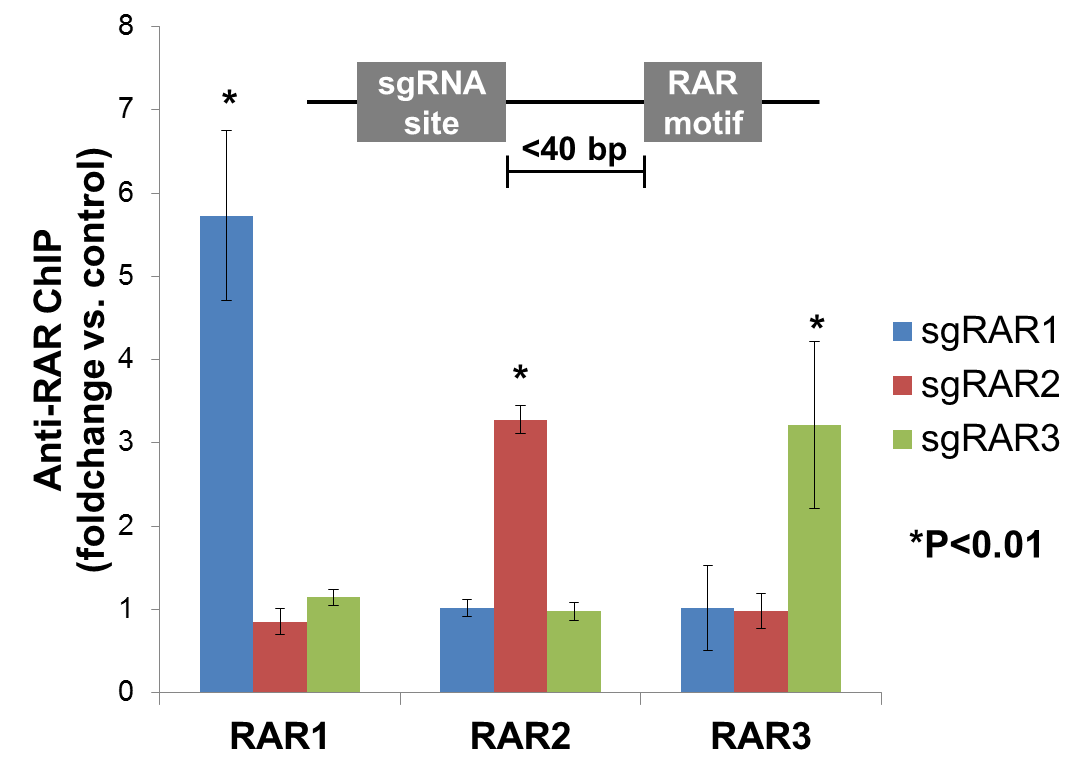

Supplement: S4 Fig — Anti-retinoic acid receptor (RAR) ChIP followed by qPCR at three loci (RAR1-3, x-axis) in the presence of sgRNAs targeting each locus (blue, red, and green). ChIP-qPCR values are normalized to control ChIP without sgRNA. Three replicates were performed for all experiments, and a two-tailed Student’s t-test was used to calculate significance, and values with P<0.01 are denoted with a *. (PNG) [file pone.0152683.s004.png]

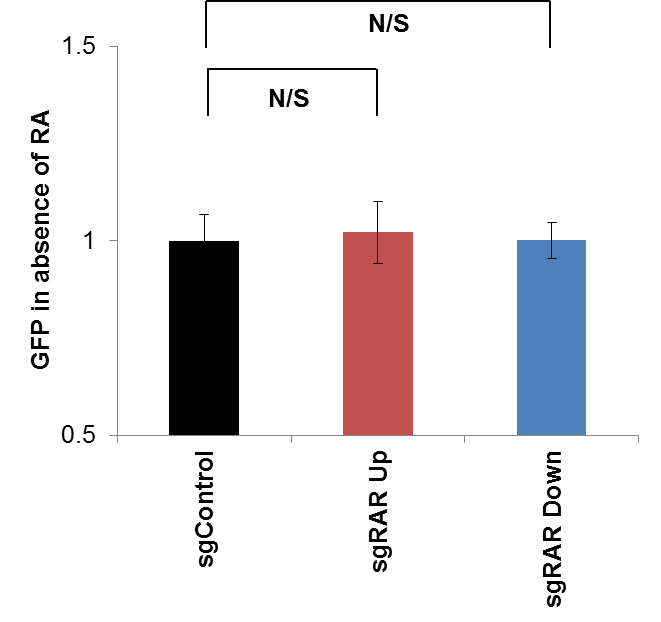

Supplement: S5 Fig — GFP expression in Tol2 RAR-GFP reporter cells in the absence of RA, normalized to cells without sgRNA. The presence of sgRNA recruiting dCas9 upstream (sgRAR up) or downstream (sgRAR down) of the RAR site has no measurable effect on GFP expression in the absence of RA. (PNG) [file pone.0152683.s005.png]
