# Supplementary material for: Cas9 Functionally Opens Chromatin
Source: PLoS One. 2016 Mar 31;11(3):e0152683. doi: 10.1371/journal.pone.0152683 (PMC4816323; doi:10.1371/journal.pone.0152683)
Supplement: S1 Table — (DOCX) [file pone.0152683.s006.docx]

**S1 Table. Oligonucleotides used in this work.**

| **CRISPR guide RNA sequences (sgRNAs used in RAR ChIP assay labeled sgRAR1-3, and sgRNAs used in the spatial mapping experiments in Figure 1c are shaded in gray)** | |
| --- | --- |
| sgRAR1 | GAGCAGGTGACAATTTCAGA |
| sgRAR2 | GTTCTGGGCAAAGCAATAAAA |
| sgRAR3 | GTGTTAATCTGGTGTTAACC |
| sgRNA4 | GACTGATTCAAAGAGGACAA |
| sgRNA5 | GCCGCACCTATCACCTGTCA |
| sgRNA6 | GCATCACAAAATGTGGGACTG |
| sgRNA7 | GGGCTTTCTGACCAGCTCGA |
| sgRNA8 | GGCTTCAGCAATGTTTTCCG |
| sgRNA9 | GCATTGCAGAGAGTCAAATT |
| sgRNA10 | GCCTTTGCAGGCATTTGTCA |
| sgRNA11 | GTGGAAAAGTGTCTTGATTGC |
| sgRNA12 | GAACTTCTACCCCTGTCAAAG |
| sgRNA13 | GCTATGGAGATATAAGCTTG |
| sgRNA14 | GTGACATCCGCCCTGAAGC |
| sgRNA15 | GAGTATCACAGAACGGGTGC |
| sgRNA16 | GTTAAGCTCACAGTCATATGC |
| sgRAR Up | GTTATTCCCTGACATAACGAA |
| sg RAR Down | GATGTTCCTGGAGCTCGCCT |
| sgControl | GGTAGTCCGGGATGTCAGCC |
| **DNase control primers** | |
| NegDNase50b_1_fw | TTGACTGCTCCCAGGTAGAGA |
| NegDNase50b_1_rv | TCTTGGTGATTTCATTCATAGGC |
| NegDNase50b_2_fw | TCCATAATGATTTGGGGAAAG |
| NegDNase50b_2_rv | GAAAGTTCTGGAAGACAGTGCAT |
| NegDNase50b_3_fw | CCAACTGCCTCCATTAGAGC |
| NegDNase50b_3_rv | TGCATGCTTGTGAATGTCAA |
| PosDNase50b_2_fw | TTTGGAAACAACCACAGTGC |
| PosDNase50b_2_rv | CAATACGCAGCTTTGACCAG |
| PosDNase50b_4_fw | GTTAAACCCAGCCTCAGTGG |
| PosDNase50b_4_rv | CTTCCAGGGCCTTCTTTGAT |
| PosDNase50b_5_fw | TTCAGGGTCCAAATAGCAGTC |
| PosDNase50b_5_rv | TGTTGTTAGAATGGCCACCA |
| **ChIP control primers (negative control primers are the same as DNase negative control primers)** | |
| Rar_Cyp26a1_fw | CCTGCAGGGGTCACAGG |
| Rar_Cyp26a1_rv | TTCCCAATCCTTTAGCCTGA |
| Rar_Rarb_fw | ACCTTCAAATGACCCAACCA |
| Rar_Rarb_rv | TGGGAGTTGGTGATGTCAGA |
| **DNase-qPCR primers for sgRNA accessibility experiments in Figure 1b. sgRAR1-3 primers also serve as ChIP-qPCR primers in Figure 2a.** | |
| sgRAR1_fw | TGCTTTCAGAGCAGGTGACA |
| sgRAR1_rv | CCAACAAAGCTCCCTCCTTC |
| sgRAR2_fw | CCTTCTGGGCAAAGCAATAAA |
| sgRAR2_rv | ATAGTGGGAGGAGCGAGAAG |
| sgRAR3_fw | TGAGAGTGACCTCTCTGTGACC |
| sgRAR3_rv | CAAGAATGTTTCCAATAACTGTGC |
| sgRNA4_fw | ATAGTGGGAGGAGCGAGAAG |
| sgRNA4_rv | AGAAAATCTGTAGCGGCCGT |
| sgRNA5_fw | TCATTTATTCCCTGACATGGTCT |
| sgRNA5_rv | TGGCCAATCCTTGAGACATT |
| sgRNA6_fw | TGTGGTGAGAAGCACCTTTG |
| sgRNA6_rv | AGGGGTCAAGGAGAGGTCAC |
| sgRNA7_fw | CTAAGCTGCTGCATGACCTC |
| sgRNA7_rv | GAGAGAACCTTAAGAAAGGGTCAG |
| sgRNA8_fw | GACTCTGTTGCTGGGCTTCA |
| sgRNA8_rv | TGGAACCTCGGAAAACATTGC |
| sgRNA9_fw | CAGTTTAGTGCACCAAATTTGAC |
| sgRNA9_rv | AATGGCATTGCAGAGAGTCA |
| sgRNA10_fw | CAGGCATTTGTCAGGGAATAA |
| sgRNA10_rv | CAACAAGCTGCTGCTTCAGT |
| sgRNA11_fw | TAAGCCCGCAATCAAGACAC |
| sgRNA11_rv | ACCCACCCAATAACAGTCTTCT |
| sgRNA12_fw | GCTTCATGGAGAGTGGCAGT |
| sgRNA12_rv | TGACAGGGGTAGAAGTTCAGC |
| sgRNA13_fw | AGAGATAACAGACCCGGGGC |
| sgRNA13_rv | TCACCACTTGCATCTCTCTTCT |
| sgRNA14_fw | AGCCTGCACACCTGGGCTAC |
| sgRNA14_rv | TGCTCTGAGATGCTTGCTTTC |
| sgRNA15_fw | CAGTTGCTGTGATGGATAGACA |
| sgRNA15_rv | GTAGCCCAGGTGTGCAGGCT |
| sgRNA16_fw | CTATCGCCTGGGTCAGAGAG |
| sgRNA16_rv | CGCTTTAGAATAGACCGTGACC |
| **Additional DNase-qPCR primers for sgRNA spatial mapping experiments in Figure 1c.** | |
| sgRAR1_b_fw | TTCAGAAGGAGGGAGCTTTG |
| sgRAR1_b_rv | GAGGGTCATGGAGAGGTCAG |
| sgRAR1_c_fw | AACCCCTGACCTCTCCATGA |
| sgRAR1_c_rv | CTGTGAGGCTGGTGGAAGAC |
| sgRAR1_d_fw | GTCTTCCACCAGCCTCACAG |
| sgRAR1_d_rv | CGCCAACAGATGTGAGGTCA |
| sgRAR1_e_fw | CTCACATCTGTTGGCGGTGA |
| sgRAR1_e_rv | GGACCTTATCTCCCTCTCGC |
| sgRAR1_f_fw | GCTTCCTCATGCTCTTGTGG |
| sgRAR1_f_rv | GAAATTGTCACCTGCTCTGAAAG |
| sgRAR1_g_fw | TCTACCACACTTCCAGCAGG |
| sgRAR1_g_rv | TGAGAATGAGTGTCTGGACCAG |
| sgRAR2_b_fw | CTTCTCGCTCCTCCCACTAT |
| sgRAR2_b_rv | CGTGAGACAAGGGAGCTGA |
| sgRAR2_c_fw | TCCCTTGTCTCACGATCTGTTT |
| sgRAR2_c_rv | AACAAACACCAGGGCCAGTT |
| sgRAR2_d_fw | AACTGGCCCTGGTGTTTGTT |
| sgRAR2_d_rv | CAGGTCAAAGGGCACTGGAG |
| sgRAR2_e_fw | GCTTCAGGGGTGAATTCAGAT |
| sgRAR2_e_rv | CATTTTATTGCTTTGCCCAGAAG |
| sgRAR2_f_fw | CAGAGGCAGCATTTAGAAGG |
| sgRAR2_f_rv | TGTATCTGAATTCACCCCTGA |
| sgRAR2_g_fw | TTTAGAATTAGCTGCAGACGG |
| sgRAR2_g_rv | AGCCTTCTAAATGCTGCCTCT |
| sgRAR2_h_fw | ACTTCATTTCTGTGTGCATGATTT |
| sgRAR2_h_rv | CCCTGTGATGCTGAGTTGTAAA |
| sgRNA4_b_fw | TGATAACATTGATGCCTTGTCCT |
| sgRNA4_b_rv | CAAATTAAGTCTTTCTACTCACAACA |
| sgRNA4_c_fw | TGATTCAAAGAGGACAAGGGCA |
| sgRNA4_c_rv | CCCTGGAACTCAAAGCTCGT |
| sgRNA4_d_fw | ACGAGCTTTGAGTTCCAGGG |
| sgRNA4_d_rv | CGGCCGAGAACAGAAAACAA |
| sgRNA4_e_fw | TGTTCTCGGCCGCCTTCG |
| sgRNA4_e_rv | CACAATAATAATAATTTATCACGAACGC |
| sgRNA4_f_fw | CCCACCTAGAAGAATTGGAGA |
| sgRNA4_f_rv | ACGGCCGCTACAGATTTTCT |
| sgRNA4_g_fw | CGCGTTCGTGATAAATTATTATTATTGT |
| sgRNA4_g_rv | AGATTCATCCATCTGCTCCACA |
| sgRNA5_b_fw | GGGACAAACACCCAAGAGAA |
| sgRNA5_b_rv | TATGAAGACCCCAGCCAGAC |
| sgRNA5_c_fw | TGTTTTCCAAGCAGCTGAAG |
| sgRNA5_c_rv | CACACATATTATGAAAACACCAACA |
| sgRNA5_d_fw | TAATGGACAGATGGCCTTGG |
| sgRNA5_d_rv | CTCAGCTTTGTCCTTGAGCA |
| sgRNA5_e_fw | GAGCATTGTTCCCGGATTAC |
| sgRNA5_e_rv | CGGAGCCAACCATCAAAG |
| sgRNA5_f_fw | AGGGCTGGGGCTTTACTTTA |
| sgRNA5_f_rv | GCTGGCAGCTCTCTGCAT |
| sgRNA5_g_fw | CGCTCTTCACCTGAATAAAATG |
| sgRNA5_g_rv | TTTGCCCAAATCCTTCTGTT |
